# Ultrasensitive Detection of MicroRNA in Human Saliva via Rolling Circle Amplification Using a DNA-Decorated Graphene Oxide Sensor

**DOI:** 10.1021/acsomega.3c00411

**Published:** 2023-04-17

**Authors:** Piyawat Pitikultham, Thitirat Putnin, Dechnarong Pimalai, Nuankanya Sathirapongsasuti, Chagriya Kitiyakara, Qiao Jiang, Baoquan Ding, Deanpen Japrung

**Affiliations:** † CAS Key Laboratory of Nanosystem and Hierarchical Fabrication, CAS Center for Excellence in Nanoscience, National Center for Nanoscience and Technology, Beijing 100190, China; ‡ School of Nanoscience and Technology, University of Chinese Academy of Sciences, Beijing 100049, China; § National Nanotechnology Center, National Science and Technology Department Agency, Thailand Science Park, Pathumthani 10120, Thailand; ∥ Program in Translational Medicine, Chakri Naruebodindra Medical Institute, Faculty of Medicine Ramathibodi Hospital, Mahidol University, Bang Pli, Samutprakarn 10540, Thailand; ⊥ Department of Medicine, Faculty of Medicine, Ramathibodi Hospital, Mahidol University, Bangkok 10400, Thailand

## Abstract

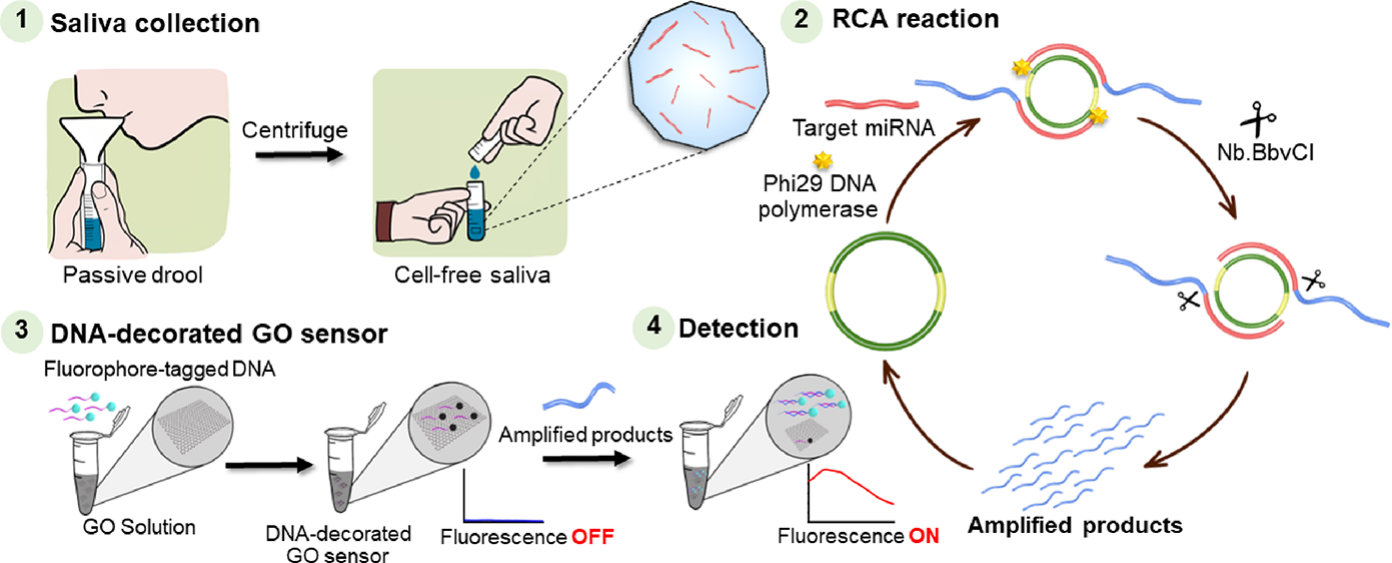

MicroRNAs (miRNAs) are a family of conserved small noncoding RNAs whose expression is associated with many diseases, including cancer. Salivary miRNAs are gaining popularity as noninvasive diagnostic biomarkers for cancer and other systemic disorders, but their use is limited by their low abundance and complicated detection procedure. Herein, we present a novel self-assembly approach based on rolling circle amplification (RCA) and graphene oxide (GO) for the ultrasensitive detection of miRNA21 and miRNA16 (miRNA oral cancer biomarkers in human saliva). First, target miRNA hybridizes with the RCA template. In the presence of DNA polymerase, the RCA reaction is induced and sequences matching the template are generated. Then, a nicking enzyme cuts the long ssDNA product into tiny pieces to obtain the amplified products. The DNA-decorated GO sensor was fabricated by preabsorbing the ssDNA fluorescence-labeled probe on the GO surface, resulting in fluorescence quenching. The DNA-decorated GO sensor could detect the amplified product via the self-assembly of dsDNA, leading to the desorption and recovery of the fluorescence-labeled probe. Under optimal conditions, the proposed system exhibited ultrasensitive detection; the detection limits of miRNA16 and miRNA21 were 8.81 and 3.85 fM, respectively. It showed a wide range of detection between 10 fM and 100 pM for miRNA16 and between 10 fM and 1 nM for miRNA16. It demonstrated high selectivity, distinguishing between 1- and 3-mismatch nucleotides in target miRNA. Overall, our proposed DNA-decorated GO sensor can accurately detect the salivary miRNAs and may potentially be used for the diagnosis and screening of early-stage oral cancer.

## Introduction

MicroRNAs (miRNAs) are small single-stranded RNA molecules of 21–24 nucleotides that play crucial roles in various biological processes, including gene expression, cell proliferation, and apoptosis. Recent research has found that changes in miRNA correlate to multiple diseases, such as cardiovascular diseases,^1^ neuronal diseases,^2^ and cancer.^3−5^ miRNAs can also be found in biological fluids, such as serum, plasma, whole blood, and saliva, at low concentrations.^6^ As specific miRNAs can alter oncogenes and tumor suppressor genes, they are emerging as prospective biomarkers for cancer diagnosis.^7^ Hence, miRNAs can be used as biomarkers for cancer detection.^8^ For example, an increase in miRNA17–92 levels is associated with a decrease in the tumor suppressor gene levels,^9,10^ and a change in miRNA21 levels in peripheral blood can be linked to the progression of oral squamous cell carcinoma.^11^ Salivary miRNA21 levels in patients with mucosal lesions and those in patients with normal mucosa are significantly different.^12^ miRNA21 expression levels are upregulated in patients with severe dysplasia.^13^ Significant progress has been made in determining the origin and functions of miRNAs and their prospective uses in research and therapeutic settings. Therefore, miRNAs may serve as effective diagnostic biomarkers.

The current gold standard for miRNA analysis is based on direct reverse transcription-polymerase chain reaction (RT-PCR) and direct RT-based and poly(A) tailing-based SYBR miRNA assays. They are time-consuming, error-prone, and intricate procedures and require professional assistance.^14,15^ Northern blotting is an alternative technique that involves the separation of the whole quantity of RNA on a polyacrylamide gel and subsequent transfer to a nylon membrane; hence, this process is laborious and requires a significant amount of RNA.^16^ Because of these problems, attempts have been made to improve the existing methods to use less RNA and shorten the time required for miRNA detection.

Saliva testing is an innovative technique that may provide valuable information for the prognosis and clinical diagnosis of oral and systemic illnesses. This procedure is convenient due to the ease of clinical sample collection, and it is noninvasive, painless, simple, and does not require expert help.^17−19^ Several molecular indications of local and systemic diseases, including cancer, have been identified via diagnostic investigations of the saliva. Owing to their exceptional stability and resistance to degradation, miRNAs in saliva have emerged as good biomarker candidates for cancer in recent years.^20^ However, identification of miRNAs at low concentrations is a challenge, and the close sequence homology among miRNA families makes it difficult to distinguish them. New assays have mainly focused on target miRNA amplification to increase the sensitivity and selectivity of the method owing to the need for rapid miRNA profiling.^3^ Several approaches, including nanoparticle-based detection,^21^ strand displacement assay,^22^ quantum-dot-based detection,^23^ colorimetric based detection,^24^ nanopore sensor-based detection,^25^ electrochemistry,^26^ and isothermal rolling circle amplification (RCA), have been developed.^27−29^ Hence, development of a new method to measure the low levels of circulating miRNAs in saliva or other biological fluids is very important for the early detection and diagnosis of cancer.

RCA is an isothermal amplification reaction for viral DNA and RNA replication.^30^ Using DNA polymerase, hybridization between the circular template and primers or target miRNAs can trigger the creation of lengthy repeating ssDNA sequences around the template. Millions of copies of the products containing specific regions are generated exponentially. Consequently, signaling and detection efficiency increase and sensitivity of the assay is enhanced.^31^ Because RCA enhances the specificity and sensitivity of detection, without the need of any sophisticated equipment, it has become one of the most efficient ways to analyze miRNAs.

The specificity of RCA-based methods can be enhanced by combining with other techniques. For example, clustered regularly interspaced palindromic repeat (CRISPR)/CRISPR-associated protein 9 can be used to cut specific regions and activate the RCA reaction.^32^ The G-quadruplex can be designed as an RCA product that specifically binds to hemin.^29^ Hyperbranched RCA is another well-known method. The system includes a second or third set of primers that hybridizes with the RCA products, enabling further amplification in less time.^33,34^ Previous studies combined RCA and DNA nicking reactions to form a short single-stranded amplicon for the activation of secondary amplification. In contrast to the conventional RCA reaction, the nicking-based RCA reaction can be exponentially accelerated once the nicking fragments form tiny multiple copies of primers.^35^ However, other reports showed that a system-nicking-based RCA response may not display secondary amplification if the initial amplicon RCA is much greater than the padlock probe. Tian et al. created a padlock probe ligation-based approach for distinguishing drug-resistant *Mycobacterium tuberculosis* (Mtb) mutations utilizing a real-time opto-magnetic sensor.^36^ The device can identify target DNA with a detection limit as low as 15 fM.^36^ As mentioned above, the RCA-base reaction is well suited for detecting low-abundance miRNAs in biological fluids, such as plasma and saliva.

Graphene oxides (GOs), sheets of planar carbon atoms, are promising nanomaterials owing to their unique properties, including a high surface area to volume ratio, high mechanical strength, excellent electrical conductivity, and strong fluorescence quenching activity.^37^ They are extensively used in biomedical applications, including biosensing, drug delivery, and cell imaging. GO-based fluorescent sensors can determine the concentration of target molecules by observing the relationship between fluorescence quenching and recovery. GOs containing π-rich conjugation domains can form π–π stacking interactions with the nucleotide bases of ssDNA and RNA but less interaction with dsDNA.^38^ Based on their quenching ability, GOs have been applied in fluorescence resonance energy transfer or fluorescence-based biosensor applications for the detection of various biomarkers, such as DNA,^39^ miRNAs,^28^ and proteins.^40^

In this study, for the first time, we combined RCA with a DNA-decorated GO sensor for highly sensitive miRNA detection in saliva samples. First, a padlock probe with a predetermined design was introduced into the ligation template to form the circularized template. The phosphate groups of the 5′ and 3′ backbones were then joined by T4 DNA ligase to generate the RCA template. Exonuclease I was used to digest the remaining primers throughout the operation. Subsequently, the RCA template and phi29 DNA polymerase were introduced into the target miRNA to create long concatemer ssDNA. The entangled RCA product was hydrolyzed into many amplified products complementary to DNA-coated GO by the nicking endonuclease. Self-assembly of amplified fragments and preabsorbed fluorescence-labeled probes on the GO surface release and recover the fluorescence response proportional to the target miRNA concentration. In our application design, we would like to utilize the simple miRNA quantification method; thus, we have reduced the process by creating a circular template that avoids the requirement for ligation process and using subtraction method to reduce the false positive.

Hence, our biosensor can discriminate single miRNA mismatches in the actual salivary samples.

## Experimental Section

### Materials and Chemicals

All oligonucleotides and fluorescence-labeled ssDNA probes (Table 1) were high-performance liquid chromatography grade and purchased from Integrated DNA Technologies, Ltd. (Singapore). Phi29 DNA polymerase, T4 DNA ligase, exonuclease I (Exo I), dNTPs, Nb. BbvCI, RNAse inhibitor, and their corresponding buffers were obtained from New England Biolabs. Inc. (England). SYBR Gold was purchased from Thermo Fisher Scientific (USA). GO powder was synthesized using a modified Hummers’ method, as described in our previous study.^40^

**Table 1:** Oligonucleotide Sequences Used in This Study^a^

oligonucleotides	sequences (5′–3′)
miRNA21	UAGCUUAUCAGACUGAUGUUGA
miRNA16	UAGCAGCACGUAAAUAUUGGCG
miRNA155	UUAAUGCUAAUCGUGAUAGGGG
miRNA29a	UAG CAC CAU CUG AAA UCG GUU A
padlock probe 21 (PP21)	phosphate–CTGATAAGCTA**CCTCAGC**TCAACATCAGTCTGATAAGCTA**CCTCAGC**TCAACATCAGT
padlock probe 16 (PP16)	phosphate-ACGTGCTGCTA**CCTCAGC**CGCCAATATTTACGTGCTGCTA**CCTCAGC**CGCCAATATTT
F-21	Cy5-CATCAGTCTGATAAGC
F-16	FAM-CAATATTTACGTGCTG
miRNA21-mm1	UAGCUUAUCgGACUGAUGUUGA
miRNA21-mm3	UuGCUUAUCgGACUGAUcUUGA
miRNA21-nc	GUAAGGCAUCUGACCGAAGGCA

a Circularized hybridization and nicking regions are underlined and bold, respectively. Mismatched nucleotides are shown in lowercase.

### Circularized Template Preparation

Circularized DNA probes were prepared by ligation. The ligation reaction mixture (20 μL) consisted of 2 μL of 100 nM miRNA21, 2 μL of 100 nM padlock probe 21, and 12 μL of ultrapure and RNase-free water. The mixture was heated to 95 °C and gradually cooled for 1 h for annealing. Next, 2 μL of 4 U/μL T4 DNA ligase and 2 μL of 10× ligation buffer were added. The ligation mixture was incubated at 37 °C for 2 h and heated at 65 °C for 10 min to terminate the enzyme activity. Subsequently, 1 μL of Exo I (20 U/μL) was added to the ligation mixture, incubated at 37 °C for 30 min, and heated at 80 °C to inactivate Exo I. The ligation reaction mixture was stored at 4 °C until further use.

### RCA

An aliquot of 20 μL of RCA reaction mixture containing 10 μL of ligation reaction mixture, and 2 μL of 10× phi29 buffer solution (500 mM Tris–HCl, 100 mM MgCl_2_, 100 mM (NH_4_)_2_SO_4_, and 40 mM DTT [pH 7.5]) was mixed with 1 μL of bovine serum albumin (BSA, 2 mg/mL), 1 μL of 10 mM dNTP, 1 μL of phi29 DNA polymerase (10 U/μL), 0.5 μL of Nb.BbvCI (10 U/μL), 2 μL of target miRNA, and 2.5 μL of deionized water. The RCA reaction mixture was then incubated at 30 °C for 2 h. The RCA reaction was terminated by heating the mixture at 65 °C for 10 min.

### Gel Electrophoresis Analysis of RCA Products

To analyze the ligation and RCA product, the 10% denatured polyacrylamide gel electrophoresis solution (PAGE) was prepared with 2 mL of 10× tris/borate/EDTA (TBE) solution, 2.5 mL 40% acrylamide/bisacrylamide, 9.6 g urea, 200 μL of 10% ammonium persulfate (APS), and 20 μL of TEMED. Finally, deionized (DI) water was added to a final volume of 20 mL. Subsequently, the DNA sample, padlock probe strands, ligation products, and RCA products were dyed using a purple (6×) gel loading dye. The RNA samples were stained with an RNA gel loading dye (2×). Electrophoresis was performed at 80 and 120 V for 10 and 50 min, respectively. The gels were stained with SYBR Gold for 30 min and imaged using an in vivo FX PRO imaging system (Bruker, Germany).

### Preparation of GOs

GOs were prepared using the same procedures as those reported in our previous study.^40^ First, graphite powder (3.0 g) was dissolved in a solution mixture containing 69 mL of H_2_SO_4_ and 1.5 g of NaNO_3_ under stirring at 0 °C. Next, 9.0 g of KMnO_4_ was gradually added to the solution mixture, which was then warmed at 35 °C for 30 min. DI water (500 mL) was slowly injected into the reaction solution, which was then heated at 98 °C for 15 min. The solution was then placed in a water bath for 10 min to cool. Subsequently, 420 mL of deionized (DI) water and 3 mL of H_2_O_2_ were added. A cellophane bag was used to sterilize the solution mixture. The bag was stirred in distilled water until the pH reached 7. The solution was then vacuumed and dried at 40 °C for 24 h. The GOs powder was then mashed using a mortar to obtain a fine powder. The final products were characterized by X-ray photoelectron spectroscopy (XPS) and transmission electron microscopy (TEM).

### DNA-Decorated Graphene Oxide Assay for miRNA Detection

GO powder (1–10 mg/mL) was dissolved in DI water and sonicated for at least 30 min until a homogeneous solution was obtained. DNA-decorated GO sensor was prepared by adding 5 μL of 2 μM fluorescence-labeled probe to 5 μL of GO solution (1–10 mg/mL). Mixing was performed by repetitive pipetting several times. GOs and fluorescence-labeled probes were incubated in the dark for 10 min. Subsequently, the sample was adjusted to a final volume of 100 μL with 1× phosphate-buffered saline solution and transferred to a black 96-well plate (Costar; 96-well black polystyrene plate). The final concentrations of GOs and fluorescence-labeled probes were 50–500 μg/mL and 100 nM, respectively. For sensitivity testing, 5 μL of the RCA product from various concentrations of target miRNA was introduced to the optimal concentration of the DNA-decorated GO sensor (10 mg/mL) and was further incubated in the dark for 30 min before being transferred to a black 96-well plate. The fluorescence response of the sample was measured using a fluorometer (SpectraMax, Molecular Devices, USA) at 640/670 nm for the F-21 complex (Cy5) and 492/520 nm for the F-16 complex (FAM).

### Saliva Sample Collection and Processing

Saliva samples were collected and processed according to the method described by Majem et al.^41^ Healthy subjects were controlled for oral hygiene for at least 1 h prior to collection and then mouthwashed with water to void the mouth of saliva. This study conformed to the declaration of Helsinki and was approved by the Faculty of Medicine Ramathibodi Hospital Ethics Committee (approval number COA.MURA2023/106). Unstimulated saliva was allowed to accumulate on the floor of the mouth and spill into a 50 mL centrifuge tube. Next, the saliva was centrifuged at 10,000 rpm for 15 min at 4 °C to separate it from the cellular phase. RNase inhibitor (1 μL/mL) was added to the cell-free saliva (CFS) supernatant. The solution was stored at −80 °C for further experiments.

### Recovery and Accuracy of the DNA-Decorated GO Sensor in Determining the Human Saliva Composition

To assess the recovery and accuracy of the DNA-decorated GO sensor in the actual biological component, the CFS samples of healthy volunteers were diluted 100-fold in DI water for analysis. Diluted CFS was then spiked with various amounts of synthetic miRNA16 and miRNA21 to obtain final concentrations ranging from 1 to 100 pM. The RCA reaction was conducted using protocols identical to those used in the previous experiment. We added 2 μL of the spiked CSF sample to the RCA reaction mixture instead of the buffer containing miRNA, totaling 20 μL. RCA was then performed for 2 h. Using the linear equation of each linearity range from each miRNA calibration curve, the fluorescence response of the RCA product from spiked samples was converted to concentration and compared to the specific value of the fluorescence response at each concentration to obtain the recovery percentage. Accuracy was calculated from three replicated samples.

### Synthesis of cDNA from Synthetic miRNAs

Diluted CFS containing various concentrations of spiked miRNAs was subjected to reverse transcription using protocols from the TaqMan Advanced miRNA cDNA Synthesis Kit (Applied Bio-Systems, USA), including the following processes: poly(A) tailing, adaptor ligation, reverse transcription, and miR-amplification (miR-Amp) reactions, which will be briefly discussed, for the whole 5 μL of poly(A) reaction volume comprising 2 μL of spiked miRNA CFS and 3 μL of poly(A) reaction mixture containing 0.5 μL of 10× poly(A) buffer, 0.5 μL 10 mM ATP, and 0.3 μL of poly(A) enzyme (5 U/μL). The mixture was then incubated at 37 °C for 45 min and 65 °C for 10 min. For the ligation of the adaptor, 5 μL of the poly(A) tailing product was mixed with an adaptor ligation mixture made up of 3 μL of 5× DNA ligase buffer, 4.5 μL of 50% polyethylene glycol 8000, 0.6 μL of 25× ligation adaptor, and 1.5 μL of RNA ligase (10 U/μL). The total reaction volume was 15 μL. The mixture was incubated at 16 °C for 60 min. Next, 15 μL of adaptor ligation product was added to the reverse transcription reaction mixture containing 3 μL of 10× RT buffer, 1.2 μL of 25 mM dNTP mix, 1.5 μL of 20× universal RT primer, and 3 μL of 10× RT enzyme mix in a total volume of 30 μL for the reverse transcription. The reaction mixture was incubated at 42 °C for 15 min and at 85 °C for 5 min. Finally, 5 μL of the reverse transcription product was subjected to an miR pre-amplification reaction, which contained 25 μL of 2× miR-Amp master mix and 2.5 μL of 20× miR-Amp primer mix in a total volume of 50 μL. The conditions for the miR-Amp response cycling were 95 °C for 5 min, followed by 14 cycles of 95 °C for 3 s and 60 °C for 30 s, followed by 99 °C for 10 min.

### Determination of Spiked miRNA Levels in Human Saliva Samples via Quantitative Real-Time PCR (qRT-PCR)

Spiked miRNAs (miR-16 and miR-21) were analyzed using the CFX96 TouchTM real-time PCR detection system (Bio-Rad, USA) in three individual experiments. PCR amplification was carried out using the TaqMan Fast Advanced Master mix kit (Applied Biosystems) according to the manufacturer’s instructions. In brief, 5 μL of miR-Amp reaction products were combined with 10 μL of 2× TaqMan Fast Advanced Master mix, 2 μL of 20× TaqMan Advanced miRNA Assay, and 4 μL of RNAase-free water in a total volume of 20 μL. PCR was performed at 95 °C for 20 s, followed by 50 cycles of 95 °C for 1 s and 60 °C for 20 s. Linearity of the qRT-PCR assay was assessed using the cycle threshold (Ct) of five tenfold serial dilutions of miRNA16 and miRNA21. Then, the linearity range was used to define the recovery% of RT-qPCR and compared with that of the DNA-decorated GO sensor.

## Results and Discussion

### Principle of DNA-Decorated GO Sensor

**Scheme 1:**
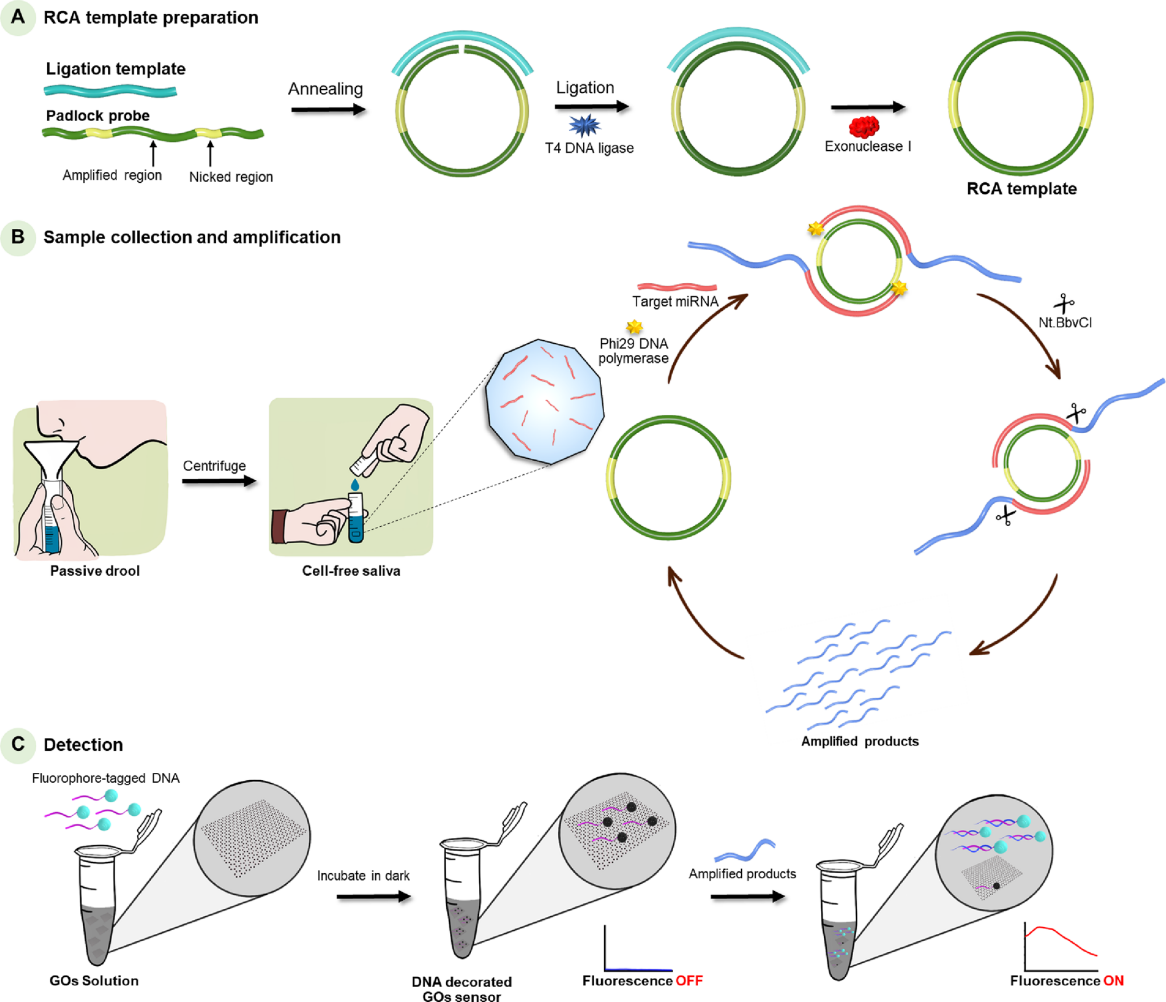
Schematic Representation of the Fabrication of the DNA-Decorated Graphene Oxide (GO) Sensor (A) Preparation of the rolling circle amplification (RCA) template via the ligation reaction. (B) Sample collection and amplification via RCA reaction. (C) DNA-decorated GO sensor can detect the amplified products based on the self-assembly of amplified products and fluorescence-labeled probe.

The system was designed to amplify the target miRNA and detect it using fluorescence response, as shown in Scheme 1. First, we designed the sequences of the linear padlock probes to have a specific complementary binding region at both sides of the terminal ends (5′ and 3′ ends) toward the ligation template. Padlock probes can form a circularized template after hybridization with the ligation template. T4 DNA ligase was added to the system to connect the 5′ end of linear ssDNA containing a phosphate group and the 3′ end. Next, Exo I was added to the mixture to digest the left ligation template after the ligation reaction. The RCA reaction was performed to amplify the low quantity of miRNAs in the saliva. The circularized templates and the target miRNAs were complementarily bound before adding to the RCA solutions containing phi29 DNA polymerase and dNTP, causing the production of concatemer sequences in parallel with the circularized template. The lengthy ssDNA products were nicked into tremendously amplified fragments. The ssDNA-decorated GO sensor was fabricated using self-assembly of the ssDNA fluorescence-label probe on the GO surface via the pi-stacking interaction. Because GOs are potent quenchers, the fluorescence signal moves away when the fluorophore is closed. The RCA reaction produced amplified fragments with sequences matching the fluorescent ssDNA on the GO surface. When dsDNA is formed, fluorescence-labeled probes are released from the surface of GOs, allowing the fluorescence response to return in a sense that is proportional to the amount of target miRNA. Therefore, the intensity of the fluorescence recovery can be used to analyze the target miRNA concentration.

### Assay Feasibility

**Figure 1:**
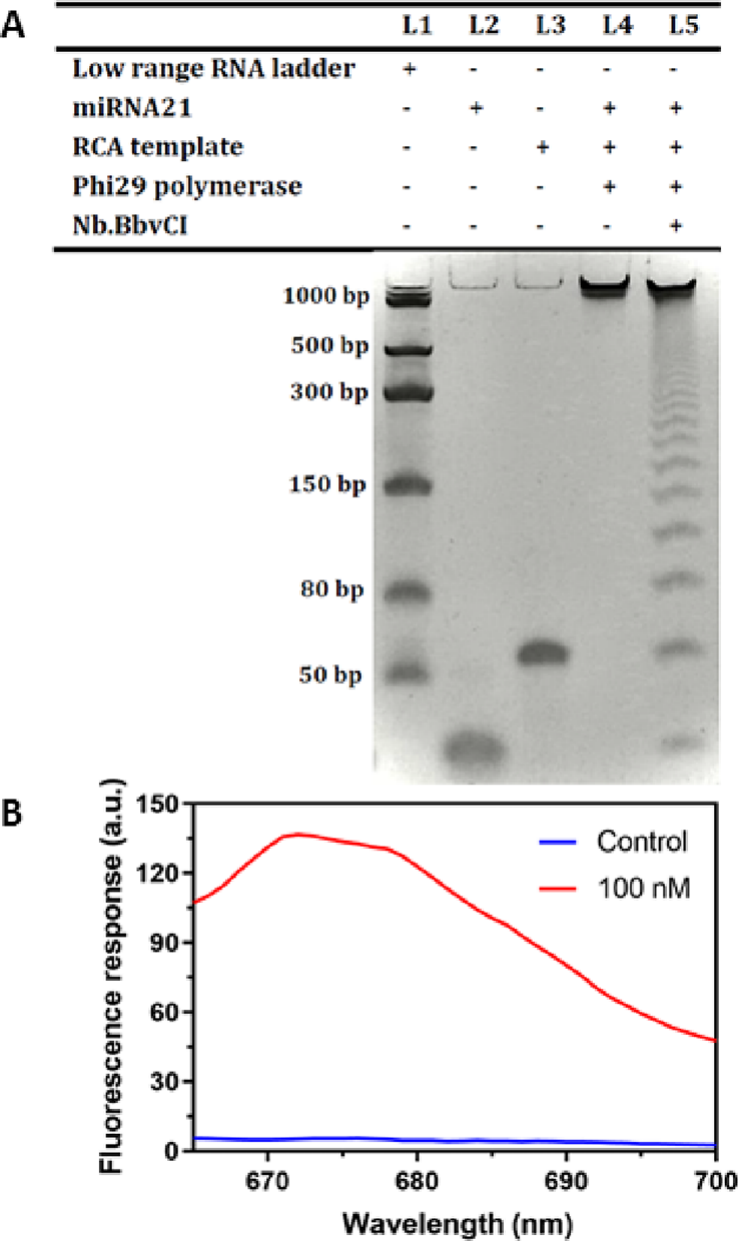
(A) 10% polyacrylamide gel electrophoresis (PAGE) of low range RNA ladder (lane 1), 100 nM microRNA (miRNA)-21 (lane 2), 100 nM padlock probe 21 (lane 3), and the product of DNA-decorated graphene oxide (GO) sensor without and with Nb.BbvCI (lane 4 and lane 5, respectively). (B) Fluorescence response of the amplification product by DNA-decorated GOs in the presence (red) and absence (blue) of miRNA21. The reaction solution contained 100 nM miRNA21 and 100 nM padlock probe 21. Ligation reactions were performed at 24 °C for 2 h, and rolling circle amplification (RCA) reactions were performed at 30 °C for 2 h.

The feasibility of this biosensor for miRNA21 detection was determined using fluorescence quenching and recovery properties and electrophoretic analysis. The RCA reaction can produce long concatemer ssDNA repeating sequences in the presence of all assay components, including the target miRNA, padlock probe (circular template), phi29 DNA polymerase, and Nb.BbvCI. All products were confirmed by 10% urea-PAGE; Figure S4 displays the ligation product before and after exonuclease I treatment. The absence and presence of nicking enzyme are shown in Figure 1A, and fluorescence spectral measurements are shown in Figure 1B. The presence of the target miRNA, the circularized template, and phi29 polymerase can generate long-concatenated DNAs, which are large molecules with high molecular weights, so they tend to migrate slightly from the well of the gel (lane 4 in Figure 1A). The anti-strand of ssDNA from the RCA reaction was designed to have a nicking region divided into short fragments by Nb.BbvCI. We observed multiple bands of DNA products of different base pairs in length, which indicated the production of nicked fragments of the RCA reaction (lane 5, Figure 1A). For further validation of the RCA reaction, we adjusted the concentration of miRNA21 and monitored the generation of the nicking fragment to compare the presence and absence of the target miRNA in the RCA process. The concentration of miRNA-21 varied between 0 and 10 nM. The results are shown in Figure S5, where the significant changes in the quantities of RCA product and nicking fragment correspond to the miRNA21 concentration (as shown in Table S3). After ensuring the RCA reaction, the nicked fragments were employed in the DNA-decorated GO sensor to induce the desorption of the fluorescence-labeled probe on the GO surface. The recovery of the fluorescence response is shown in Figure 1B. In the presence and absence of the target miRNA, the DNA-decorated GO sensor turned on and off in the fluorescence response. These results illustrate the feasibility of using this sensor and nicked fragments for signal amplification.

### Optimization of DNA-Decorated GO Sensor Assay

**Figure 2:**
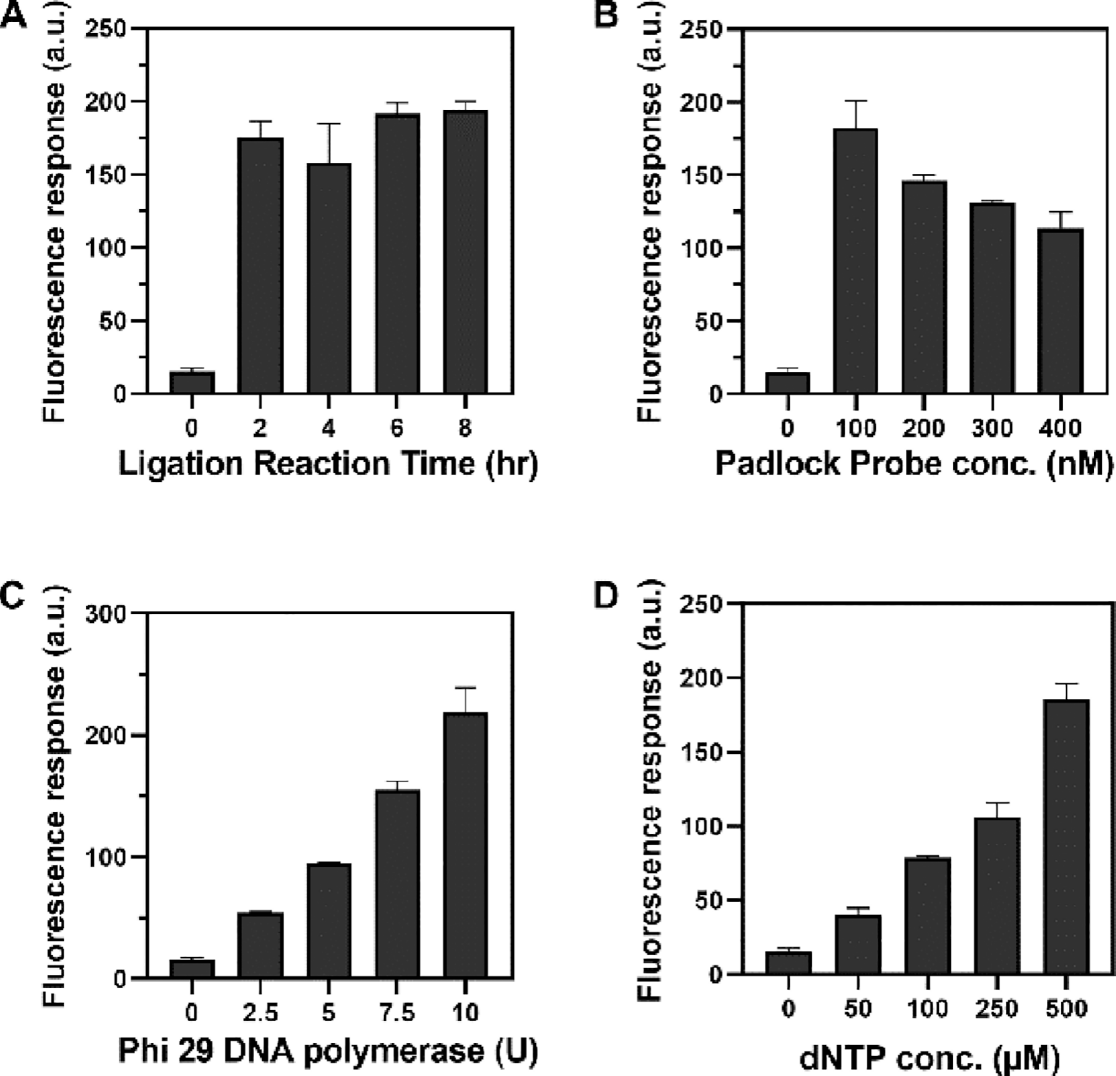
Fluorescence response of the sensing system under different conditions: (A) variation of ligation reaction time, (B) different concentrations of padlock probe concentration, (C) different amounts of phi29 DNA polymerase, and (D) different concentrations of dNTPs.

To achieve the best performance of this assay, we optimized numerous dependent components of the RCA reaction, including the reaction time in the ligation process, padlock probe concentration, phi29 DNA polymerase, and dNTP concentration. The ligation reaction time was varied from 2 to 8 h; however, there was no significant change in the fluorescence intensity response (Figure 2A). Thus, we chose a 2 h ligation reaction time for subsequent experiments. We then investigated the influence of linear padlock probe concentration by varying the concentration of the padlock probe from 100 to 400 nM, while the annealing process containing 100 nM target miRNA utilized circularized template formation. As shown in Figure 2B, the fluorescence response of the system decreased slightly with increasing linear padlock probe concentration. The preabsorbed fluorescence-labeled probe and padlock probe had identical complementary sequences for the nicked product. As a result, both may establish competitive binding, causing a reduction in the signal response once padlock probe concentrations are high. The concentration of phi29 DNA polymerase varied from 2.5 to 10 U; the fluorescence intensity increased with phi29 polymerase (Figure 2C). The RCA product was a long ssDNA that was assembled by a nucleotide monomer; therefore, dNTP was an essential supply for the RCA reaction. As shown in Figure 2D, the fluorescence intensity increased with increasing dNTP concentration, and the highest signal was obtained at 500 μM. Hence, the 2 h ligation reaction, 100 nM padlock probe, 10 U of phi29 DNA polymerase, and 500 μM dNTP were selected as the optimal concentrations for our developed platform.

### Optimization of Self-Assembly of the Fluorescence-Labeled Probe on GO Surface

The adsorption and desorption of ssDNA fluorescence-labeled probes on the GO surface can be used for biochemical analyses. This mechanism is dependent on many factors, such as DNA length, pH, and salt concentration. However, ssDNA showed affinity toward the GO surface via hydrogen bonding and π–π stacking interactions. As a result, ssDNA fluorescence-labeled probes can be adsorbed onto GOs, enabling the quenching of the fluorescence signal.^38,42^ Meanwhile, the reaction could be reversed after adding the complementary, which detached the ssDNA from the GO surface.

GO was synthesized by modifying the Hummer’s method from a previous study.^40^ X-ray photoelectron spectroscopy (XPS) was used to characterize the functional groups of the GO powder. The XPS survey scan of GOs showed apparent peaks at binding energies of approximately 530–535 and 284–288 eV, which indicated the existence of oxygen (O 1s) and carbon components (C 1s), respectively. This result also correlated with the previous study of another group that the peak of O 1s was higher than the peak of C 1s in GOs.^43^ The deconvoluted C 1s spectra of GOs (Figure S1) showed an increase in the binding energies at 285.00, 287.194, and 288.745 eV, which were C–C, C–O, and C=O, respectively. The GO morphology was observed using TEM (Figure S2). The image shows a thin GO sheet, which was expected to exhibit a single-layer structure. The images confirm that GOs were successfully synthesized.

**Figure 3:**
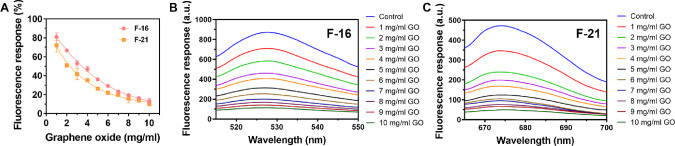
Fluorescence quenching of fluorescence-labeled probes (F-16 and F-21) in different concentrations of GOs. (A) Fluorescence response (%) of F-16 and F-21. (B) The fluorescence response spectrum of F-16 (FAM) is 515–550 nm. (C) The fluorescence response spectrum of F-21 (Cy5) is 665–700 nm.

Our system can detect miRNA16 and miRNA21 from the RCA product using two fluorescence-labeled probes (FAM-F16 and Cy5-F21) and different padlock probes (PP16 and PP21). The ratio of GOs to fluorescence-labeled probes must be optimized to increase the signal-to-noise ratio of the sensor. The GO solution was varied in the range of 0–10 mg/mL and mixed with a 2 μM fluorescence-labeled probe (F-16 and F-21). As shown in Figure 3, the fluorescence intensity decreased with increasing GO concentration and reached 90 percent quenching at 10 mg/mL in both F-16 and F-21. Herein, 10 mg/mL of GOs was selected for the subsequent fluorescence-based sensor system.

### Sensitivity of DNA-Decorated GO Sensor

**Figure 4:**
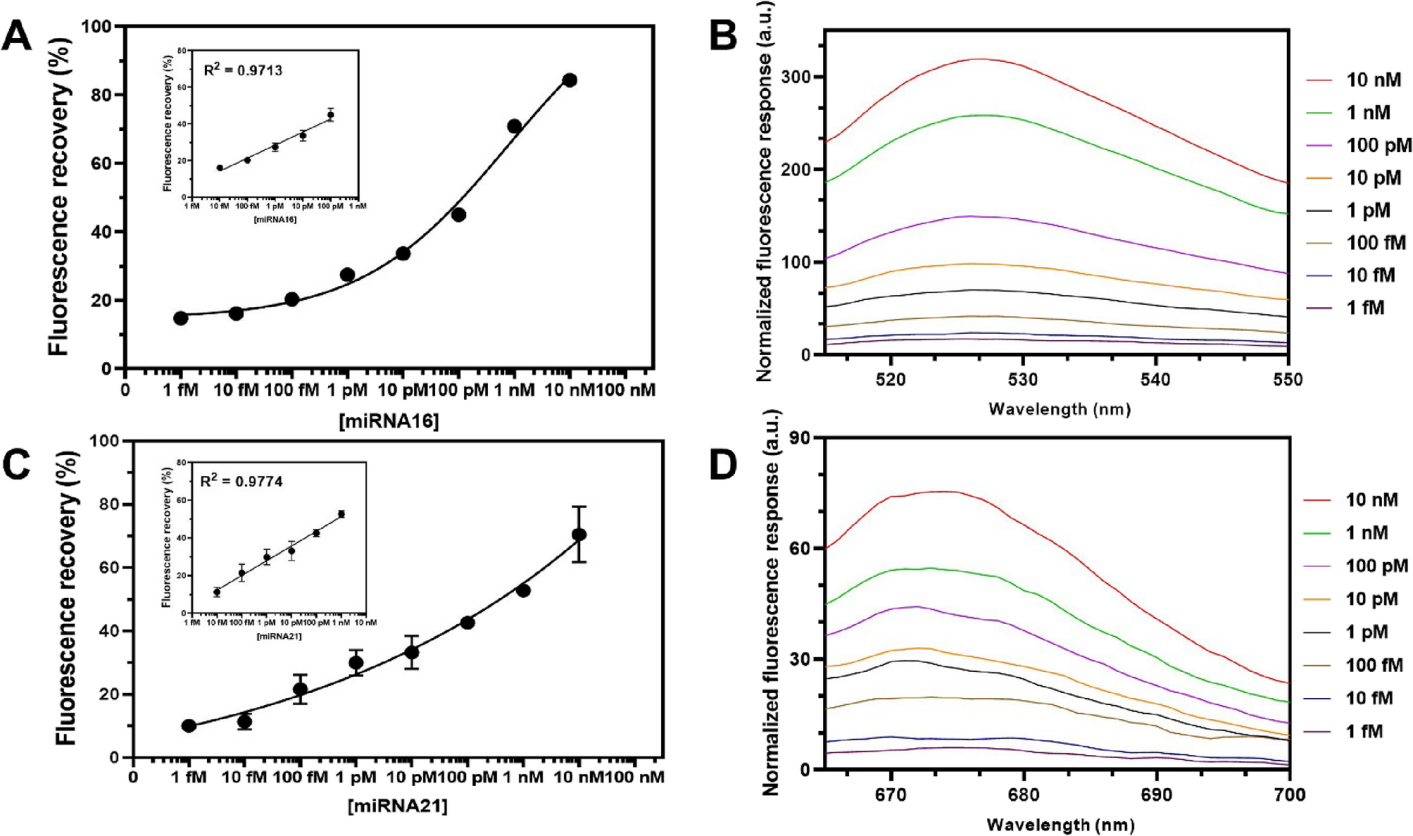
Fluorescence response of the DNA**-**decorated GO sensor at different concentrations of target miRNAs. Calibration curve and linearity range of logarithm miRNA16 (A) and miRNA21 (C) concentrations. Fluorescence response spectrum from 0 to 10 nM for miRNA16 (B) and miRNA21 (D).

To demonstrate the sensitivity of the DNA-decorated GO sensor, we analyzed the RCA products from various concentrations of target miRNAs (miRNA21 and miRNA16) under optimal conditions (Figure 4). Target miRNAs were prepared in ultrapure and RNAase-free water using the protocols described in the Experimental Section and were varied from 1 fM to 100 nM. System sensitivity was evaluated by sensing the fluorescence responses at 525 and 670 nm for miRNA16 and miRNA21, respectively. The peak of the fluorescence emission spectra increased progressively with the increase in target miRNA concentrations over a wide range from 0 to 100 nM, indicating desorption of the fluorescence-labeled probe from the GO surface. Meanwhile, the linearity correlation of the logarithm of miRNA16 and miRNA21 concentrations was found in the range of 10 fM to 100 pM and 10 fM to 1 nM, respectively. The regression equations of miRNA16 and miRNA21 were log_10_*Y* = 7.109 log[miRNA16] – 7.19 (*R*^2^ = 0.9774) and log_10_*Y* = 7.416 log[miRNA21] + 5.46 (*R*^2^ = 0.9713), where *Y* is the value of the fluorescence response. Notably, the proposed method’s limit of detection (LOD) was calculated based on the 3.3σ rule, which was 8.81 and 3.85 fM for miRNA16 and miRNA21, respectively. In addition, our developed sensor is comparable to other previous approaches for miRNA detection. The sensor has a competitive detection limit and reaction time in saliva samples (Table S1).^29,33,44−47^ This DNA-decorated GO sensor can detect miRNA16 and miRNA21 with acceptable sensitivity.

### Selectivity of DNA-Decorated GO Sensor

**Figure 5:**
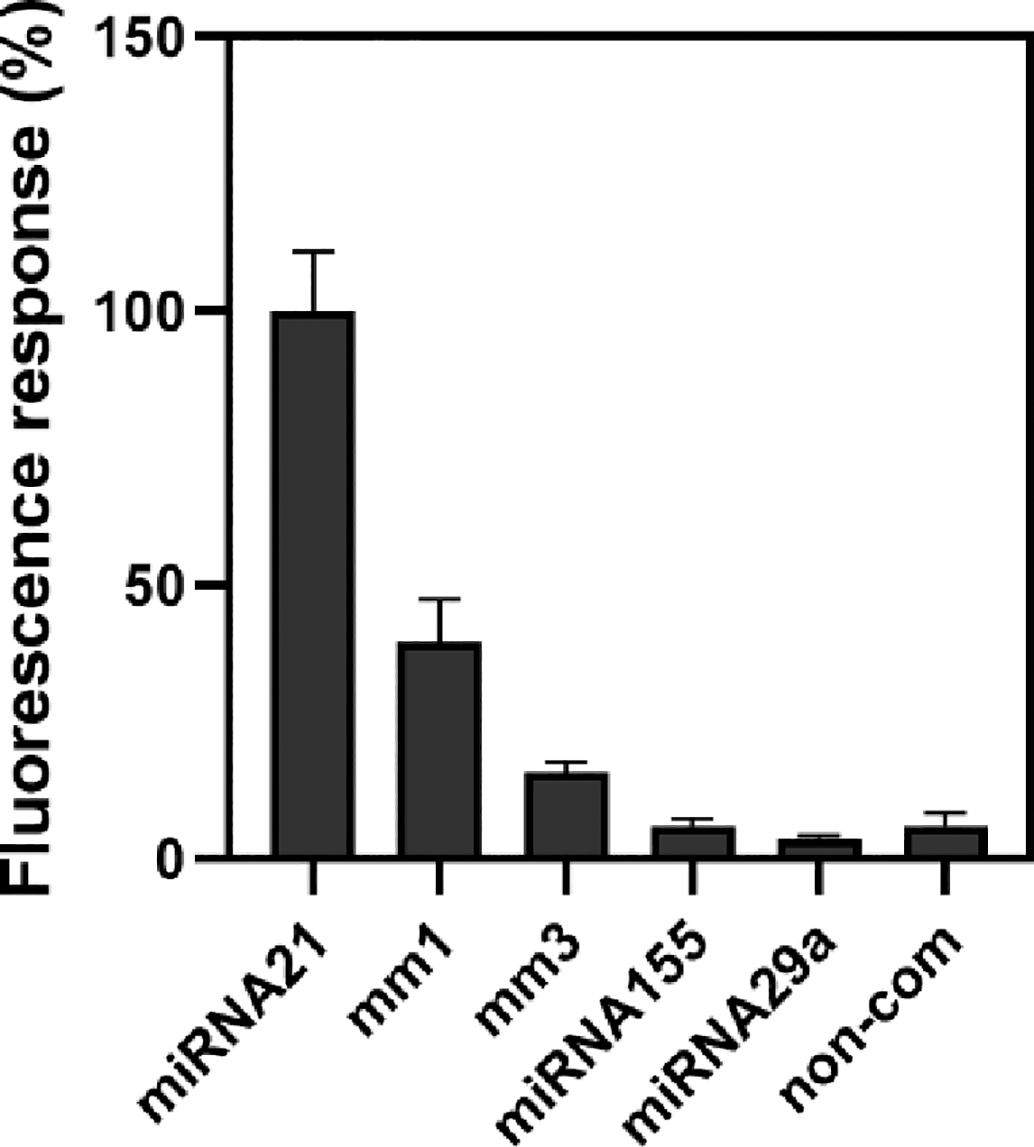
Selectivity of the DNA**-**decorated GO sensor to detect miRNA21 mismatches. Fluorescence response (%) of 10 nM miRNA21, miRNA21-mm1, miRNA21-mm3, miRNA155, miRNA29a, and noncomplementary (non-com).

Members of the miRNA family carry similar sequences.^3,4^ To evaluate the selectivity and specificity of the DNA**-**decorated GO sensor, we used one base pair mismatch miRNA21 (miRNA21-mm1), three base pair mismatch miRNA21 (miRNA21-mm3), and noncomplementary (miRNA21-nc) in this study. The fluorescence response of miRNA21 was defined as the 100% response; the results showed that the fluorescence responses of miRNA21-mm1, miRNA21-mm3, and miRNA21-nc were 39.42 ± 10.87, 16.17 ± 1.88, and 6.091 ± 2.57%, respectively (Figure 5). Furthermore, we tested the selectivity toward other types of miRNAs. The findings demonstrated that the fluorescence response to miRNA155 was 6.04 ± 1.33%, and the response to miRNA29a was 3.72 ± 0.5%. These results confirm that the DNA-decorated GO sensor can distinguish the different types of miRNAs, the single-base and three-base mismatched target sequences exhibits good selectivity for actual clinical sample analysis.

### Recovery and Accuracy of DNA-Decorated GO Sensor in Determining the Composition of Human Saliva Samples

Recently, salivary miRNAs have attracted significant interest for the noninvasive diagnosis of several diseases, especially inflammation and cancer in the oral cavity.^3,20^ For the applicability of our platform to biologically relevant complexes, spike-and-recovery tests were performed on diluted CFS samples comprising three 10-fold dilutions of miRNA16 and miRNA21 (1–100 pM). To prove the efficiency of our developed sensor, the results were compared with those of the RT-qPCR assay of miRNAs. By using the DNA-decorated GO sensor, the recovery% from the determination of spiked miRNA CFS samples was 91.1 to 106.91% and 83.17 to 121.54% for miRNA16 and miRNA21, respectively (Table 2). To compare our strategy with the gold-standard strategy, a five-fold dilution of miRNA16 and miRNA21 was utilized by RT-qPCR to determine the linear response from 10 nM to 1 pM. The cycle threshold of each replicate sample (*n* = 3) was used to generate the regression equation of spiked miRNA16 and miRNA21 in CFS samples, which were *Y* = −3.41 log[miRNA16] + 29.36 (*R*^2^ = 0.998) and *Y* = −3.07 log[miRNA21] + 27.93 (*R*^2^ = 0.998), respectively, where *Y* is the value of Ct (Figure S3). The recovery percentages of RT-qPCR, which were calculated from the Ct of the different concentrations of spiked miRNA (Table S2), were between 91.94 and 112.22% for miRNA16 and between 125.52 and 148.95% for miRNA21. Statistical analysis (two-tailed test) revealed that the *P* value for every experiment was greater than 0.05, indicating that our approach is as accurate as RT-qPCR. This confirmed that our system is reliable and effective for detecting miRNAs in CFS samples.

**Table 2:** Determination of MicroRNA (miRNA)-16 and (miRNA)-21 Levels in Spiked Cell-Free Saliva (CFS) Samples Using the DNA-Decorated Graphene Oxide (GO) Sensor and Reverse Transcription-Quantitative Polymerase Chain Reaction (RT-qPCR) Analysis (*n* = 3)

		DNA-decorated GO sensor			RT-qPCR			
samples	spiked miRNA conc (pM)	found miRNA (pM)	recovery (%)	RSD (%)	found miRNA (pM)	recovery (%)	RSD (%)	calculated *T*-test
miRNA16	100	106.92	106.92	22.41	112.22	112.22	2.03	0.35
	10	9.11	91.1	21.46	11.04	110.38	9.13	3.08
	1	0.97	96.97	26.91	91.94	91.94	19.69	0.33
miRNA21	100	83.17	83.17	17.77	140.68	140.68	22.04	2.23
	10	10.44	104.38	22.02	12.55	125.52	18.09	0.92
	1	1.22	121.54	25.86	1.49	148.95	1.15	1.50

## Conclusions

In summary, we successfully developed a novel DNA-decorated GO sensor, the miRNA16 and miRNA21 fluorescence assay, which shows high sensitivity and selectivity. The proposed method can multiply trace amounts of target miRNA in the sample into large amounts of nicking products. The LODs of our proposed sensor are 8.81 and 1.4 fM for miRNA16 and miRNA21, respectively, under optimized conditions. Our platform was able to determine the concentrations of spiked miRNA in CFS samples with acceptable RSD% and recovery% equivalent to gold-standard methods, such as RT-qPCR. DNA-decorated GO sensors require approximately 3 h for detection, from sample collection to signal readout. In contrast to RT-qPCR, miRNA-based methods involve many steps, including poly(A) tailing, adaptor ligation, reverse transcription, and miR-AMP reaction. The entire procedure may take up to 5 h. Hence, technical proficiency is required to handle the complexity of this method. Moreover, various steps involved in this process may lead to sample contamination. Because isothermal reactions do not require heat cycling, they are more suitable for use in point-of-care diagnostic equipment. More clinical evaluation studies are under investigated and will be published in the next manuscript. We also recommend utilizing Exo I and Exo III to eliminate all traces of the ligation template from the circular template; this will reduce false positives and improve the consistency of the detection result. In conclusion, our DNA-decorated GO system for salivary miRNA detection is a unique noninvasive tool that can potentially be used in clinical settings for the early prognosis and diagnosis of oral cancer.

## Supporting Information Available

(Figure S1) X-ray photoelectron spectroscopy (XPS) results for graphene oxides; (Figure S2) transmission electron microscopy (TEM) images of graphene oxides; (Figure S3) analysis of the linearity of reverse transcription-quantitative polymerase chain reaction (RT-qPCR) results targeting spiked microRNA (miRNA)-16 and miRNA21 in the salivary sample of a healthy volunteer; (Figure S4) 10% denatured polyacrylamide gel electrophoresis (PAGE) of low range RNA ladder (lane 1), microRNA (miRNA)-21 (lane 2), padlock probe 21 (lane 3), ligation product (lane 4), and ligation product after exonuclease I treatment (lane 5); (Figure S5) 10% denatured polyacrylamide gel electrophoresis (PAGE) of RCA products from the different concentrations of miRNA21 ranging from zero (control) to 10 nM miRNA21 RCA reaction solution containing a 10 μL of circular template, 2 μL of 10x phi29 buffer, 1 μL of 2 mg/mL BSA, 1 μL of 10 mM dNTP, 2 μL of target miRNA, 2.5 μL of DI water, 1 μL of phi29 polymerase (10 U/μL), and 0.5 μL of Nb.BbvCI. (10 U/μL); (Table S1) comparison of various strategies for the quantification of microRNAs (miRNAs); (Table S2) repeatability of reverse transcription-quantitative polymerase chain reaction (RT-qPCR) amplification of miRNA16 and miRNA21 (samples 1, 2, and 3 refer to three independent analyses of each spiked miRNA cell-free saliva [CFS] sample); and (Table S3) band area/density from ImageJ analysis of Figure S5 (PDF)

## ABBREVIATIONS

APS: ammonium persulfate
BSA: bovine serum albumin
CFS: cell-free saliva
dNTP: deoxynucleotide
Exo I: exonuclease I
GO: graphene oxide
miRNA: microRNA
PAGE: polyacrylamide gel electrophoresis
RCA: rolling circle amplification
RT-PCR: reverse transcription-polymerase chain reaction
TEM: transmission electron microscopy
XPS: X-ray photoelectron microscopy
