# Supplementary material for: Ultrasensitive Detection of MicroRNA in Human Saliva via Rolling Circle Amplification Using a DNA-Decorated Graphene Oxide Sensor
Source: ACS Omega. 2023 Apr 17;8(17):15266–75. doi: 10.1021/acsomega.3c00411 (PMC10157686; doi:10.1021/acsomega.3c00411)
Supplement: Supplementary file 1 — ao3c00411_si_001.pdf [file ao3c00411_si_001.pdf]

# Ultrasensitive Detection of MicroRNA in Human Saliva via Rolling Circle Amplification Using a DNA-Decorated Graphene Oxide Sensor

Piyawat Pitikultham[1,2], Thitirat Putnin[3], Dechnarong Pimalai[3], Nuankanya Sathirapongsasuti[4], Chagriya Kitiyakara[5], Qiao Jiang[1,2], Baoquan Ding[1,2], and Deanpen Japrung[3]*

[1]CAS Key Laboratory of Nanosystem and Hierarchiral Fabrication, CAS Center for Excellence in Nanoscience, National Center for Nanoscience and Technology, Beijing 100190, China

[2]School of Nanoscience and Technology, University of Chinese Academy of Sciences, Beijing 100049, China

[3]National Nanotechnology Center (NANOTEC), National Science and Technology Department Agency (NSTDA), Thailand Science Park, Pathumthani, 10120, Thailand

[4]Program in Translational Medicine, Chakri Naruebodindra Medical Institute, Faculty of Medicine Ramathibodi Hospital, Mahidol University, Bang Pli, Samutprakarn, 10540, Thailand.

[5]Department of Medicine, Faculty of Medicine, Ramathibodi Hospital, Mahidol University, Bangkok 10400, Thailand

**Characterization of graphene oxide (GO)**

**X-ray photoelectron spectroscopy (XPS)**

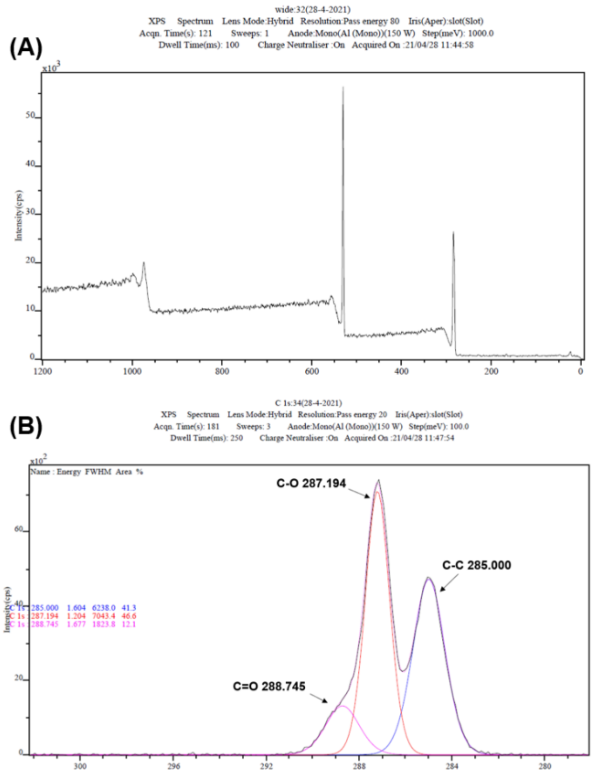


**Figure S1.** (A) X-ray photoelectron spectroscopy (XPS) survey scan of graphene oxides (GOs) and (B) deconvoluted C1s spectra of GOs.

**Transmission electron microscopy (TEM)**

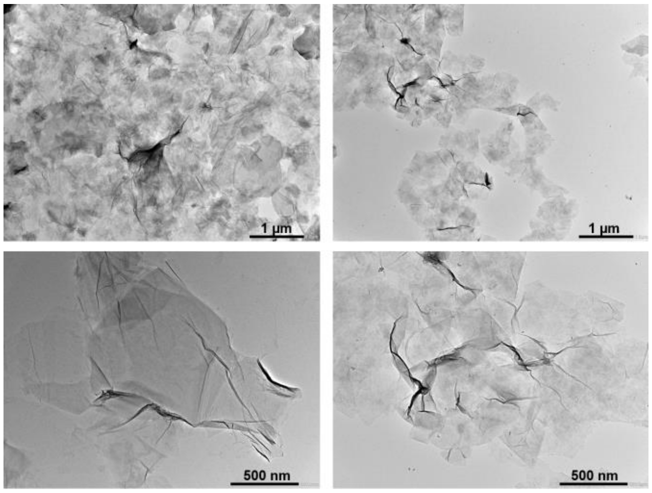


**Figure S2.** Transmission electron microscopy (TEM) images of graphene oxides (GOs).

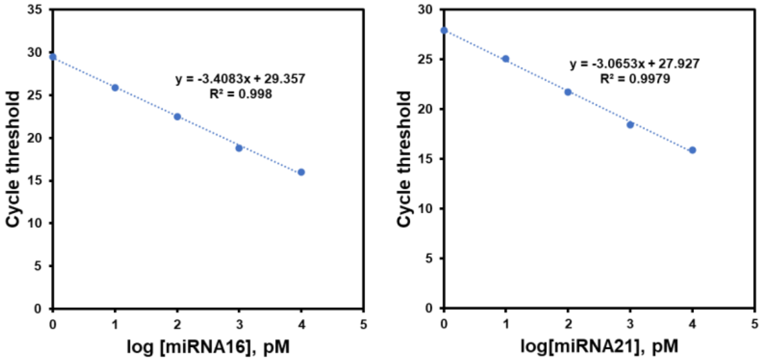


**Figure S3.** Analysis of the linearity of reverse transcription-quantitative polymerase chain reaction (RT-qPCR) results targeting spiked microRNA (miRNA)-16 and miRNA21 in the salivary sample of a healthy volunteer.

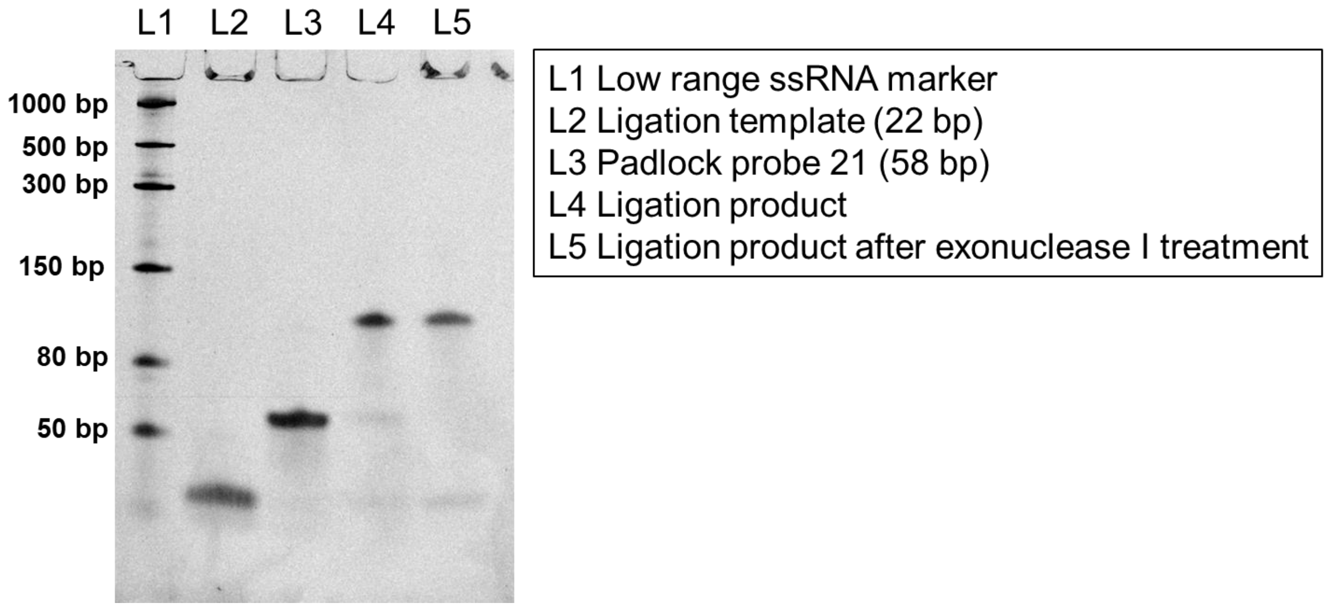


**Figure S4.** 10% Denatured polyacrylamide gel electrophoresis (PAGE) of low range RNA ladder (lane 1), microRNA (miRNA)-21 (lane 2), padlock probe 21 (lane 3), ligation product (lane4), and ligation product after exonuclease I treatment.

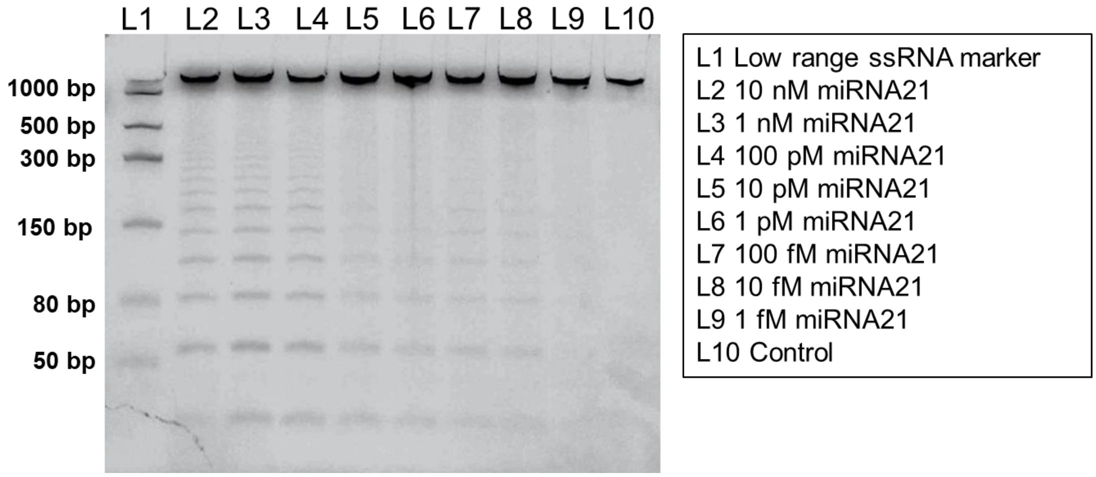


**Figure S5.** 10% denatured polyacrylamide gel electrophoresis (PAGE) of RCA products from the different concentrations of miRNA21 ranging from zero (control) to 10 nM miRNA21 RCA reaction solution containing a 10 μL of circular template, 2 μL of 10x phi29 buffer, 1 μL of 2 mg/mL BSA, 1 μL of 10 mM dNTP, 2 μL of target miRNA, 2.5 μL of DI water, 1 μL of phi29 polymerase (10 U/μL), and 0.5 μL of Nb.BbvCI. (10U/μL).

**Supplementary Tables**

| Spiked Conc. | miRNA16 100 pM | miRNA16 10 pM | miRNA16 1 pM | miRNA21 100 pM | miRNA21 10 pM | miRNA21 1 pM |
|---|---|---|---|---|---|---|
| Sample 1 | 22.34 | 25.67 | 29.19 | 21.06 | 24.32 | 27.41 |
| Sample 2 | 22.4 | 25.81 | 29.56 | 21.39 | 24.61 | 27.38 |
| Sample 3 | 22.37 | 25.94 | 29.75 | 21.64 | 24.79 | 27.4 |
| Average | 22.37 | 25.81 | 29.5 | 21.36 | 24.57 | 27.4 |
| Std | 0.03 | 0.14 | 0.28 | 0.29 | 0.24 | 0.015 |

**Table S1.** Comparison of various strategies for the quantification of microRNAs (miRNAs).

**Table S2.** Repeatability of reverse transcription-quantitative polymerase chain reaction (RT-qPCR) amplification of miRNA16 and miRNA21 (samples 1, 2, and 3 refer to three independent analyses of each spiked miRNA cell-free saliva [CFS] sample).

| Method | Amplification strategy | Target | Sample | LOD | Reaction time | Refs. |
|---|---|---|---|---|---|---|
| Fluorescence | Nicking-enhanced RCA and quantum dots | Let-7a | Serum | 4.6 fM | 5.30 hr. | (29) |
| Fluorescence | Branched RCA | miRNA21 | Serum | 1 pM | 5 hr. | (33) |
| Fluorescence | RCA and CRISPR/Cas12a | miRNA21 | Cell | 34.7 fM | 3.30 hr. | (44) |
| Fluorescence | RCA and $WS_2$ nanosheet | miRNA21 | Buffer | 300 fM | 5 hr. | (45) |
| Fluorescence | RCA and designed H probe | let-7b-5p | Serum | 10 pM | 4.30 hr. | (46) |
| Dynamic light scattering | RCA and AuNPs aggregation | Let-7a | Cell | 0.11 fM | 9 hr. | (47) |
| Fluorescence | Nicking-enhanced RCA and GO | miRNA21 | Saliva | 1.4 fM | 2.40 hr. | This work |

In conclusion, the decorated graphene oxide sensor in this work demonstrates competitive performance in terms of detection limit, reaction time, and saliva testing.

**Table S3**. Band area/density from ImageJ analysis of Figure S5.

| **Lane name** | **Band area** | |
|---|---|---|
| | **Digested RCA fragment area** | **Undigested area** |
| L2 10 nM miRNA21 | 25,332.44 | 32,315.087 |
| L3 1 nM miRNA21 | 23,461.2 | 33,033.602 |
| L4 100 pM miRNA21 | 18,727.25 | 23,128.489 |
| L5 10 pM miRNA21 | 13,732.25 | 27,945.602 |
| L6 1 pM miRNA21 | 15,254.73 | 32,358.238 |
| L7 100 fM miRNA21 | 13,145.54 | 28,817.196 |
| L8 10 fM miRNA21 | 13,019.71 | 30,814.338 |
| L9 1 fM miRNA21 | 9,525.489 | 23,721.539 |
| L10 Control miRNA21 | 2,605.154 | 15,471.761 |
